# Causal interplay between trigeminal neuralgia and systemic inflammatory markers: A bidirectional Mendelian randomization study

**DOI:** 10.1097/MD.0000000000047243

**Published:** 2026-01-23

**Authors:** Lingsen Hou, Lingbo Hou, Jianwen Guo, Jinzhou Guo, Shanshan Cui

**Affiliations:** a Department of Rehabilitation Medicine, Zhengzhou Anorectal Hospital, Zhengzhou, China; b Academy of Zhongjing, Henan University of Chinese Medicine, Zhengzhou, China; c State Key Laboratory of Traditional Chinese Medicine Syndrome, Guangzhou University of Chinese Medicine, Guangzhou, China.

**Keywords:** causal inference, chemokines, inflammatory factors, Mendelian randomization, neurotrophic factors, trigeminal neuralgia

## Abstract

Trigeminal neuralgia (TN) is a debilitating neuropathic pain disorder with profound impacts on quality of life. Although alterations in circulating inflammatory proteins have been reported, observational findings remain inconsistent and are vulnerable to confounding and reverse causation. Whether these immune changes are causal or merely downstream consequences of TN remains uncertain, underscoring the need for robust causal inference methods. We conducted a bidirectional 2-sample Mendelian randomization (MR) analysis to investigate the causal relationships between circulating inflammatory proteins and TN. Genetic instruments for 91 proteins and TN were obtained from large-scale publicly available genome-wide association studies. Five complementary MR estimators were applied – inverse-variance weighting (IVW), MR-Egger, weighted median, weighted mode, and simple mode – and sensitivity analyses were performed to evaluate heterogeneity, pleiotropy, and robustness. Forward MR identified 5 proteins significantly associated with TN risk: eotaxin (IVW odds ratio [OR] = 0.834, 95% CI = 0.711–0.977, *P* = .025); C-X-C motif chemokine ligand 5 [CXCL5]; IVW OR = 0.841, 95% CI = 0.726–0.974, *P* = .021); IL-20RA (IVW OR = 1.317, 95% CI = 1.032–1.679, *P* = .027); interleukin-6 (IVW OR = 1.343, 95% CI = 1.059–1.704, *P* = .015); and neurturin (IVW OR = 1.190, 95% CI = 1.004–1.410, *P* = .044). Reverse MR indicated that TN causally influenced 5 immune traits: eotaxin (OR = 0.39, 95% CI = 0.19–0.80, *P* = .010); CXCL5 (OR = 0.32, 95% CI = 0.11–0.91, *P* = .032); IL-20RA (OR = 2.07, 95% CI = 1.11–3.84, *P* = .022); interleukin-15 receptor α (OR = 0.28, 95% CI = 0.08–0.99, *P* = .048); and transforming growth factor-α (OR = 0.44, 95% CI = 0.23–0.83, *P* = .011). Notably, eotaxin, CXCL5, and IL-20RA showed bidirectional associations with TN. All signals were consistent across complementary estimators and sensitivity analyses, with no evidence of heterogeneity or horizontal pleiotropy (all *P* > .05). Our genetic evidence demonstrates a close coupling between the circulating inflammatory network and TN, providing testable leads for biomarker development and immunomodulatory strategies in TN.

## 1. Introduction

Trigeminal neuralgia (TN) is an excruciating orofacial neuropathic pain syndrome characterized by sudden, brief paroxysms of intense, electric shock–like pain within the distribution of the trigeminal nerve, most often unilateral.^[1]^ Attacks are commonly precipitated by innocuous stimuli – such as chewing, speaking, or light tactile contact with the face – and most frequently involve the maxillary (V2) or mandibular (V3) divisions,^[2]^ leading to a profound reduction in quality of life. The prevailing pathological substrate is a neurovascular compression at the trigeminal root entry zone – typically from an aberrant arterial loop – which induces focal demyelination and consequent hyperexcitability of affected fibers.^[3,4]^ This demyelination is thought to promote ectopic impulse generation and ephaptic cross-talk between adjacent axons (the “ignition hypothesis”), thereby producing the hallmark shock-like attacks of TN.^[3]^ Nonetheless, TN has a complex and incompletely resolved etiology. Beyond mechanical compression, converging evidence suggests that neuroinflammatory and reparative processes contribute to disease biology: activated Schwann cells can phagocytose damaged myelin and secrete neurotrophic factors, implicating immune–inflammatory signaling as a potential modulator of demyelination and neuronal excitability in TN.^[5]^

Inflammation and immune mediators have increasingly been implicated in chronic neuropathic pain states, including TN.^[6,7]^ Pro-inflammatory cytokines such as tumor necrosis factor-α (TNF-α) and interleukin-6 (IL-6) are known to enhance nociceptive signaling and foster neuropathic pain hypersensitivity.^[8]^ In trigeminal pain models, TNF-α has been linked to heightened pain responses, and other cytokines like IL-1β and IL-6 have been shown to exacerbate pain via peripheral and central sensitization mechanisms.^[2]^ Another study reported that the serum levels of IL-1β, IL-6, IL-8, and TNF-alpha in TN patients were significantly higher than those of controls and that IL-6 concentrations were positively correlated with TN severity.^[9]^ A recent study noted that the C-reactive protein to albumin ratio – an index of systemic inflammatory burden – was increased in TN patients, suggesting a more pronounced inflammatory response in this condition.^[10]^ Such findings support the notion that inflammation and cytokine dysregulation may play a role in TN pathogenesis or pain modulation. Despite these associations, conventional observational studies cannot establish whether inflammation is a cause or consequence of TN.^[11]^ Patients with severe chronic pain might exhibit elevated inflammatory markers as a result of stress and neuroendocrine changes (reverse causation), or there may be confounding factors (e.g., age, comorbid conditions, or lifestyle factors) that influence both inflammation and TN risk. These issues make it difficult to determine whether inflammatory alterations are causal contributors to TN or secondary consequences of the disease. As a result, the directionality and causality of the relationship between TN and systemic inflammation remain unresolved, representing an important knowledge gap. This uncertainty gives rise to the hypothesis that certain inflammatory proteins may exert causal effects on TN, whereas others may simply reflect downstream immune responses. To address this gap, robust analytical approaches that minimize confounding and reverse causation are required to allow reliable causal inference.

Mendelian randomization (MR) is a genetic epidemiology approach that leverages genetic variants as instrumental variables (IVs) to assess causal effects of an exposure on an outcome.^[12]^ Because alleles are assorted randomly at conception (in accordance with Mendel’s laws), MR analysis is inherently less prone to confounding and is not affected by reverse causation. Recent large-scale proteomic studies and genome-wide association analyses have further provided robust genetic instruments for a wide range of circulating inflammatory proteins,^[13]^ enabling their integration into MR frameworks for causal inference. A bidirectional MR framework further enables the evaluation of causal effects in both directions – testing whether circulating inflammatory proteins influence the risk of TN, and whether TN itself induces alterations in systemic inflammatory markers. By comparing causal estimates across both directions, this design helps distinguish true etiologic drivers from downstream biological consequences, overcoming a key limitation of traditional observational studies in which the temporal and causal order cannot be clearly defined. In this study, we apply a 2-sample bidirectional MR analysis to explore the potential causal relationships between TN and systemic inflammatory markers. Our objective is to determine whether inflammation has a causal influence on TN onset and severity, and/or whether TN has a downstream causal impact on systemic inflammation, thereby shedding light on the interplay between immune-mediated processes and the pathophysiology of TN.

## 2. Materials and methods

### 2.1. Study design

**Figure 1.**
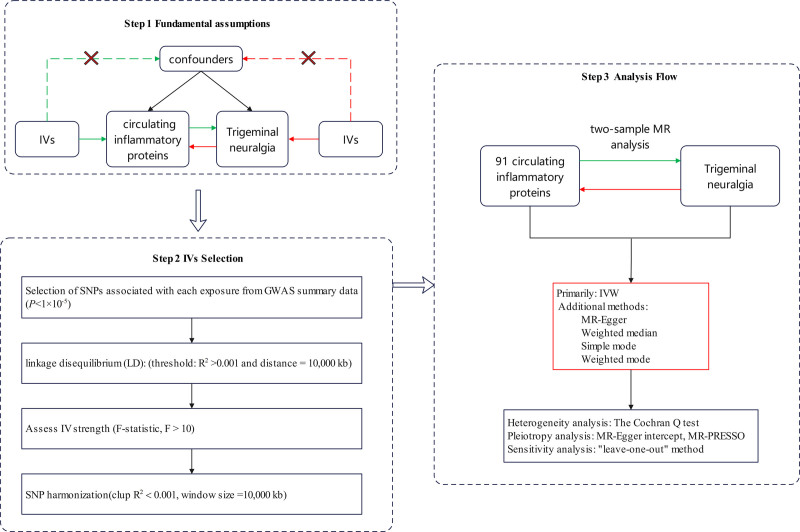
Overview of the bidirectional Mendelian randomization (MR) analysis workflow. Green arrows represent the forward MR analyses evaluating the causal effects of 91 circulating immune factors on the risk of trigeminal neuralgia. Red arrows represent the reverse MR analyses assessing whether genetic liability to trigeminal neuralgia causally influences circulating immune factor levels. IV = instrumental variable, IVW = inverse-variance weighting, LD = linkage disequilibrium, MR = Mendelian randomization.

The present study employed a 2-sample MR approach to investigate the causal relationships between 91 circulating inflammatory proteins and TN. To ensure the robustness of causal inference, IVs were required to adhere to 3 fundamental assumptions: each IV must establish direct connections with the exposure factors; each IV should not be correlated with potential confounding variables lying between the exposure and the outcome; and each IV should not influence the outcome through a pathway unrelated to the exposure.^[14]^ All MR analyses were conducted using publicly available summary statistics, thereby obviating the necessity for additional ethical approval or informed consent. The analytical principles and procedures are illustrated in Figure 1.

### 2.2. Data source

Genome-wide association study (GWAS) summary statistics for immune traits were retrieved from the GWAS Catalog; specifically, data for 91 circulating inflammatory proteins were taken from a meta-analysis of 14,824 European-ancestry participants (GWAS Catalog accessions GCST90274758–GCST90274848).^[15]^ More details were described in Table S1, Supplemental Digital Content, https://links.lww.com/MD/R233. GWAS summary statistics for TN were obtained from the FinnGen database (https://finngen.fi/), with GWAS ID finngen_R11_G6_TRINEU, comprising 397,805 individuals (case 1973; control 395,832).

### 2.3. Instrumental variable selection

According to recent studies, in order to enhance the reliability of IVs and ensure the stability of study data and the accuracy of results, IVs need to meet the following criteria: Firstly, single nucleotide polymorphisms (SNPs) strongly associated with the exposure were selected using a significance threshold of *P* < 1 × 10⁻⁵,^[16]^ a commonly used threshold in proteome-wide MR studies when genome-wide significant variants (*P *< 5 × 10⁻⁸) are limited; secondly, To eliminate linkage disequilibrium (LD) within IVs and meet the requirements of MR analysis, IVs must satisfy the condition of *R*^2^ < 0.001 and LD = 10,000 kb^[17]^; thirdly, to ensure a robust association of selected IVs with exposure, the strength of genetic variation as IVs should be assessed using the *F*-statistic (screening criterion: *F* > 10).^[18]^ The *F*-statistic was calculated using the formula:


$$\begin{array}{l} F=\frac{\left(N-K-1\right)\cdot R^{2}}{K\cdot \left(1-R^{2}\right)} \end{array}$$

where N is the sample size in the exposure database, *K* is the number of IVs, and *R*^2^ is the proportion of variance explained by SNPs in the exposure database. The formula for calculating *R*^2^ is:


$$\begin{array}{l} R^{2}=\frac{\beta^{2}\;\cdot EAF\;\cdot \;\left(1-EAF\right)}{SE^{2}\cdot \;N} \end{array}$$

where EAF is the effect allele frequency, β is the allele effect value, N is the sample size, and SE is the standard error. Finally, when combining the exposure and outcome datasets, it is essential to eliminate incompatible alleles and palindromic SNPs.^[19]^

### 2.4. Statistical analysis

#### 2.4.1. MR analysis

We conducted a 2-sample MR analysis to evaluate the causal relationship between 91 circulating inflammatory proteins and TN. The inverse-variance weighting (IVW) estimator served as the primary analytical approach.^[13]^ To assess robustness to potential violations of MR assumptions, we additionally applied simple mode,^[20]^ weighted median,^[21]^ weighted mode, and MR-Egger regression^[22]^ and compared point estimates and confidence intervals across methods. In addition, given the large number of exposures analyzed, we applied the Benjamini–Hochberg false discovery rate (FDR) procedure to control for multiple testing, reporting both raw *P*-values and FDR-adjusted *Q*-values. All statistical analyses and data visualizations were performed in R (version 4.3.2). Univariable MR was implemented using the TwoSampleMR and MendelianRandomization packages.

#### 2.4.2. Sensitivity analysis

Between-variant heterogeneity was assessed using Cochran’s *Q* statistic; *P* > .05 was interpreted as no evidence of heterogeneity.^[23]^ MR-Egger intercept tests were used to evaluate directional (horizontal) pleiotropy, with *P* > .05 indicating no detectable pleiotropy.^[24]^ We further applied Mendelian Randomization Pleiotropy RESidual Sum and Outlier (MR-PRESSO) to detect and correct for outlier variants and residual pleiotropy, using the “MRPRESSO” R package.^[25]^ Additional robustness was evaluated via leave-one-out analyses to examine the influence of individual instruments. Diagnostic forest, scatter, and funnel plots were generated to visualize effect estimates, heterogeneity, and potential small-study or asymmetry patterns.

## 3. Results

In this study, we finally selected 2998 SNPs associated with circulating inflammatory proteins as IVs. Detailed characteristics of the SNPs are shown in Table S2, Supplemental Digital Content, https://links.lww.com/MD/R233.

### 3.1. Exploration of the causal effect of immune traits on TN

**Figure 2.**
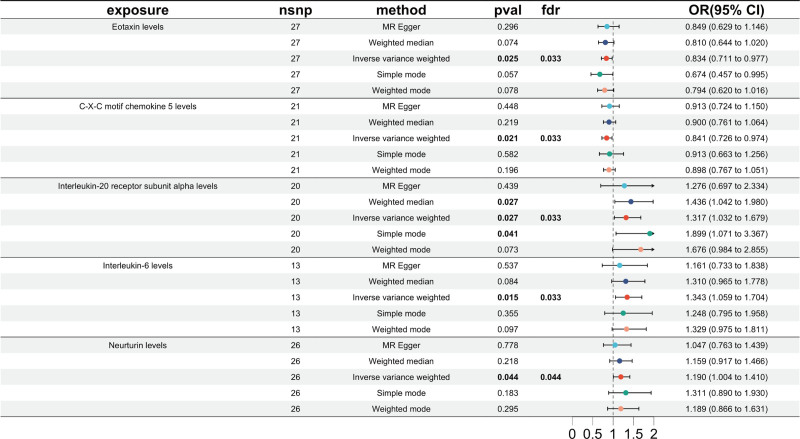
Forest plots showing significantly causal relationships between 5 circulating inflammatory proteins and TN. CI = confidence interval, nSNP = number of single-nucleotide polymorphism, OR = odds ratio, TN = trigeminal neuralgia.

**Figure 3.**
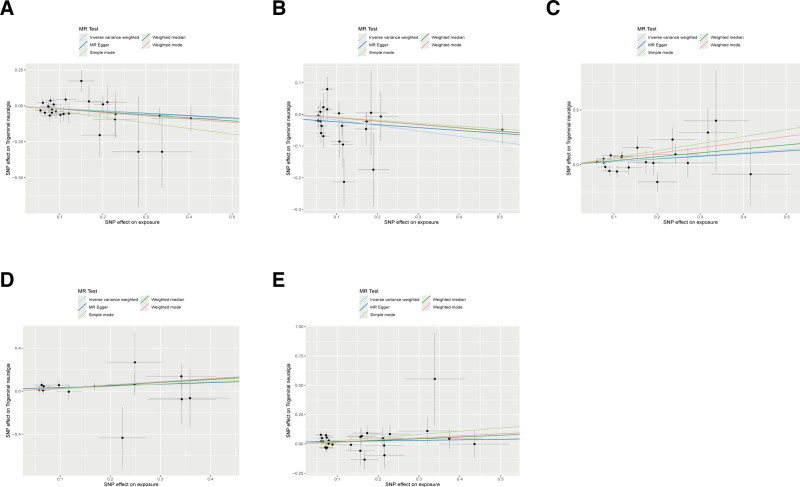
Scatter plots of causal relationships between 5 circulating inflammatory proteins and TN. (A) Eotaxin; (B) CXCL5; (C) IL-20RA; (D) IL-6; (E) Neurturin. TN = trigeminal neuralgia.

The forward MR analysis demonstrated significant causal relationships between 5 circulating inflammatory proteins and the risk of TN (Fig. 2, forest plot; Fig. 3, scatter plots). These associations were validated using the IVW method across independent datasets. Specifically, significant forward effects were observed for eotaxin (odds ratio [OR] = 0.834, 95% CI = 0.711–0.977, *P* = .025), C-X-C motif chemokine ligand 5 [CXCL5]; OR = 0.841, 95% CI = 0.726–0.974, *P* = .021), IL-20RA (OR = 1.317, 95% CI = 1.032–1.679, *P* = .027), IL-6 (OR = 1.343, 95% CI = 1.059–1.704, *P* = .015), and neurturin (OR = 1.190, 95% CI = 1.004–1.410, *P* = .044). After FDR correction, all 5 associations remained statistically significant (all *Q* < 0.05), indicating robustness to multiple testing. Four complementary MR methods (simple mode, weighted median, weighted mode, and MR-Egger) supported the direction and consistency of the IVW results. No evidence of horizontal pleiotropy or heterogeneity was detected (*P* > .05; Table 1). Additional sensitivity checks – leave-one-out (Fig. S1, Supplemental Digital Content, https://links.lww.com/MD/R234) and funnel plots (Fig. S2, Supplemental Digital Content, https://links.lww.com/MD/R234) – further confirmed robustness.

**Table 1:** Sensitivity analysis of causal relationships between 5 circulating inflammatory proteins and TN.

Exposure	Outcome	Method	Cochran’s *Q*			Pleiotropy		
			*Q*	*Q*_df	*Q*_*P*val	Egger intercept	SE	*P*val
Eotaxin	TN	MR-Egger	23.29	25	0.56	−0.0025	0.0177	.89
		IVW	23.31	26	0.62			
		MR-PRESSO						.66
CXCL5	TN	MR-Egger	24.86	19	0.17	−0.0140	0.0155	.38
		IVW	25.92	20	0.17			
		MR-PRESSO						.22
IL-20RA	TN	MR-Egger	24.65	18	0.13	0.0038	0.0339	.91
		IVW	24.67	19	0.17			
		MR-PRESSO						.20
IL-6	TN	MR-Egger	6.45	11	0.84	0.01967	0.0271	.48
		IVW	6.98	12	0.86			
		MR-PRESSO						.92
Neurturin	TN	MR-Egger	26.99	24	0.30	0.0165	0.0177	.36
		IVW	27.98	25	0.31			
		MR-PRESSO						.34

CXCL5 = C-X-C motif chemokine ligand 5, IL-6 = interleukin-6, IL-20RA = interleukin-20 receptor subunit alpha, IVW = inverse-variance weighted, MR = Mendelian randomization, MR-PRESSO = Mendelian randomization-Pleiotropy Residual Sum and Outlier, SE = standard error, TN = trigeminal neuralgia.

### 3.2. Exploration of the reverse causal effect of TN on circulating inflammatory proteins

**Figure 4.**
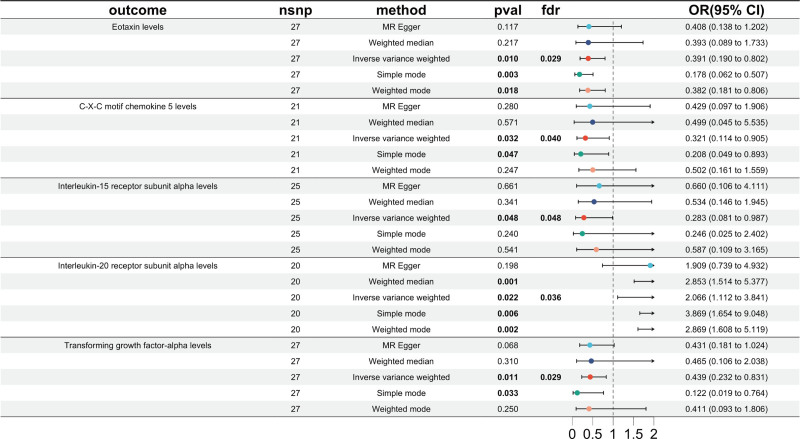
Forest plots showing causal relationships between TN and 5 circulating inflammatory proteins. CI = confidence interval, FDR = false discovery rate, nSNP = number of single-nucleotide polymorphism, OR = odds ratio, TN = trigeminal neuralgia.

**Figure 5.**
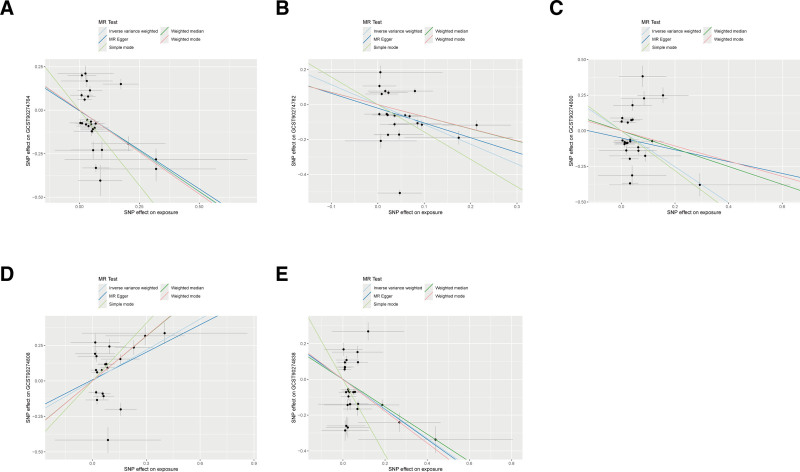
Scatter plots of causal relationships between TN and 5 circulating inflammatory proteins. (A) Eotaxin; (B) CXCL5; (C) IL-20RA; (D) IL-15RA; (E) TGF-α. TN = trigeminal neuralgia.

Reverse MR assessed whether TN causally influences circulating inflammatory proteins and identified 5 immune-related traits associated with TN as the exposure (Fig. 4, forest plot; Fig. 5, scatter plots): eotaxin (OR = 0.39, 95% CI = 0.19–0.80, *P* = .010), CXCL5 (OR = 0.32, 95% CI = 0.11–0.91, *P* = .032), IL-20RA (OR = 2.07, 95% CI = 1.11–3.84, *P* = .022), interleukin-15 receptor α [IL-15RA]; OR = 0.28, 95% CI = 0.08–0.99, *P* = .048), and transforming growth factor-α (TGF-α; OR = 0.44, 95% CI = 0.23–0.83, *P* = .011). All 5 associations also remained statistically significant after FDR correction (all *Q* < 0.05), indicating robustness to multiple testing. These findings were robust across simple mode, weighted median, weighted mode, and MR-Egger, which supported effect directions and magnitudes. No heterogeneity or horizontal pleiotropy was observed (*P* > .05; Table 2). Sensitivity analyses – leave-one-out (Fig. S3, Supplemental Digital Content, https://links.lww.com/MD/R234) and funnel plots (Fig. S4, Supplemental Digital Content, https://links.lww.com/MD/R234) – showed consistent and stable effects. Notably, eotaxin, CXCL5, and IL-20RA appeared in both directions, suggesting potential bidirectional causal relationships with TN.

**Table 2:** Sensitivity analysis of causal relationship between TN and 5 circulating inflammatory proteins.

Outcome	Exposure	Method	Cochran’s *Q*			Pleiotropy		
			*Q*	*Q*_df	*Q*_*P*val	Egger intercept	SE	*P*val
Eotaxin	TN	MR-Egger	759.41	25	0.36	−0.0032	0.0299	.92
		IVW	759.74	26	0.42			
		MR-PRESSO						.35
CXCL5	TN	MR-Egger	1216.47	19	0.21	−0.0209	0.0389	.59
		IVW	1234.99	20	0.26			
		MR-PRESSO						.67
IL-20RA	TN	MR-Egger	336.55	18	0.33	0.0079	0.0358	.83
		IVW	337.47	19	0.37			
		MR-PRESSO						.26
IL-15RA	TN	MR-Egger	1262.71	23	0.48	−0.0506	0.0410	.23
		IVW	1346.41	24	0.54			
		MR-PRESSO						.73
TGF-α	TN	MR-Egger	515.07	25	0.29	0.0015	0.0231	.95
		IVW	515.16	26	0.32			
		MR-PRESSO						.32

CXCL5 = C-X-C motif chemokine ligand 5, IL-15RA = interleukin-15 receptor α, IL-20RA = interleukin-20 receptor subunit α, IVW = inverse-variance weighted, MR = Mendelian randomization, MR-PRESSO = Mendelian randomization-Pleiotropy Residual Sum and Outlier, SE = standard error, TGF-α = transforming growth factor-α, TN = trigeminal neuralgia.

## 4. Discussion

In this study, we performed bidirectional 2-sample MR analyses to investigate the causal links between circulating inflammatory proteins and TN. Forward MR identified 5 proteins with suggestive causal effects on TN – eotaxin, CXCL5, IL-20RA, IL-6, and neurturin with consistent directions across complementary estimators and sensitivity analyses. Reverse MR further indicated that TN causally influenced 5 immune traits – eotaxin, CXCL5, IL-20RA, IL-15RA, and TGF-α. Notably, eotaxin, CXCL5, and IL-20RA were implicated in both forward and reverse directions, suggesting potential bidirectional causal relationships and a neuro–immune feedback loop in TN; taken together, the convergent signals highlight systemic inflammation (IL-6) and neurotrophic imbalance (neurturin) as upstream drivers, with TN-associated remodeling of peripheral immune/growth factor milieus (IL-15RA, TGF-α).

### 4.1. Bidirectional immune–TN interplay: eotaxin, CXCL5, and IL-20RA

Our MR analysis identified *eotaxin* (C-C motif chemokine ligand 11 [CCL11]) and *C-X-C motif chemokine ligand 5* (CXCL5) as having bidirectional causal associations with TN, suggesting a complex 2-way interplay between systemic immune factors and TN pathology.^[26,27]^ Eotaxin is a chemokine chiefly involved in recruiting eosinophils and other leukocytes; it plays important roles in inflammation and nociceptive signaling.^[28]^ In both animal models and patients, nerve injury can trigger upregulation of eotaxin/CCL11 – for instance, chronic constriction injury increases CCL11 expression in the spinal cord and dorsal root ganglia in animal models,^[26]^ and elevated CCL11 levels have been reported in the cerebrospinal fluid of patients with neuropathic pain.^[29]^ This chemokine’s recruitment of immune cells can amplify inflammatory responses at injury sites. However, eotaxin’s role in pain appears to be context-dependent. Acute increases in eotaxin may exacerbate neuroinflammation and tissue damage,^[30,31]^ whereas in later or chronic phases, eotaxin signaling might contribute to tissue repair or neuroprotective processes. This dual nature could explain our finding that genetically higher eotaxin levels protect against TN (OR < 1) while established TN tends to suppress circulating eotaxin levels. In other words, TN might down-regulate certain chemokines as a feedback mechanism, even though those chemokines (when genetically elevated) help prevent the development of TN.

CXCL5 showed a similar bidirectional pattern.^[32]^ CXCL5 (also known as epithelial neutrophil-activating peptide 78) is a CXC chemokine that attracts neutrophils and has been implicated in both inflammatory pain and healing.^[33,34]^ Notably, CXCL5 is significantly elevated in the cerebrospinal fluid of TN patients, pointing to its involvement in TN-related neuroinflammation.^[35]^ Experimentally, CXCL5 can act directly on sensory neurons: intrathecal administration of CXCL5 induces nociceptive hypersensitivity and neuropathic pain behaviors in rodent models.^[27,36]^ Conversely, CXCL5 also promotes nerve regeneration and repair; for example, it can stimulate neurite outgrowth and activate pro-regenerative pathways in Schwann cells to support axonal regrowth.^[37]^ Thus, CXCL5 functions as a double-edged sword in neuropathic conditions, facilitating both pain and recovery.^[27]^ Our MR results indicate that genetically higher CXCL5 reduces TN risk while TN onset leads to lower CXCL5 levels systemically. This could reflect that an adequate CXCL5-mediated regenerative response is protective against TN, whereas chronic TN may blunt the production or peripheral availability of CXCL5. Such a scenario aligns with literature suggesting chemokines like CXCL5 can initially drive inflammation but later assist in healing, creating a bidirectional feedback loop. Overall, the bidirectional relationships for eotaxin and CXCL5 underscore a potential vicious cycle: immune mediators influence pain development, and the pain state in turn alters immune mediator levels, which might sustain or modulate the progression of TN.

In addition, *interleukin-20 receptor subunit alpha* (IL-20RA) emerged as a bidirectionally causal factor linking immunity and TN.^[27]^ IL-20RA is part of the receptor for IL-20 and IL-24, cytokines of the IL-10 family known to drive inflammatory responses.^[38]^ We found that genetically elevated IL-20RA levels increase TN risk, and reciprocally, TN tends to upregulate IL-20RA expression or activity. This suggests a feed-forward loop between neuroinflammation and trigeminal pain. Physiologically, signaling through IL-20RA (e.g., by IL-24) can activate downstream pathways in immune and glial cells that exacerbate inflammation.^[39]^ Recent studies have demonstrated that IL-24 binding to the IL-20RA/IL-20RB receptor complex in the central nervous system provokes the production of pro-inflammatory cytokines (such as IL-1β and TNF-α) and contributes to neuropathic pain maintenance.^[40]^ Notably, nerve injury triggers upregulation of IL-24 and its receptors: for example, TNF-α stimulation leads to increased IL-24 in neurons and glial cells, and induces IL-20R expression in astrocytes and microglia.^[41,42]^ Blocking this pathway has analgesic effects – intrathecal neutralization of IL-24 or IL-20R2 (the β subunit partnering with IL-20RA) significantly alleviates neuropathic pain in animal models.^[43]^ Therefore, it is plausible that TN initiates a self-perpetuating cycle whereby the pain-induced inflammatory milieu elevates IL-20RA signaling, which in turn furthers neuroinflammation and pain.^[44]^

The absence of horizontal pleiotropy in our MR analysis supports that this is a direct effect rather than confounding. In summary, the bidirectional findings (for eotaxin, CXCL5, and IL-20RA) highlight a dynamic crosstalk: the immune system and trigeminal nerve pathology influence each other. This insight reinforces the concept that chronic neuropathic pain is not a purely neural phenomenon but involves significant immune feedback loops. Targeting such neuro-immune interactions – for example, normalizing chemokine levels or interrupting IL-20RA signaling – could be a novel approach to break the cycle of pain and inflammation in TN.

### 4.2. Upstream immune risk factors: IL-6 and neurturin

Our analysis also identified immune-related factors that exhibit a 1-way causal effect on TN risk. These upstream mediators – interleukin-6 (IL-6) and the neurotrophic factor neurturin – appear to predispose individuals to developing TN, even though TN itself did not significantly alter their circulating levels in our data. IL-6 is a prototypical proinflammatory cytokine long implicated in neuropathic pain.^[45]^ The genetic evidence linking higher IL-6 to greater TN risk reinforces and extends prior observations that IL-6 contributes to trigeminal pathophysiology. IL-6 is rapidly upregulated in injured nerves^[46]^ and can directly sensitize nociceptive neurons and activate glial cells, effectively lowering the threshold for pain initiation. In fact, a recent study using a rat chronic constriction injury of the infraorbital nerve – an established TN model – demonstrated that IL-6 levels in the cerebrospinal fluid (CSF) increase in proportion to nerve injury severity and drive astrocyte activation via STAT3 signaling, thereby exacerbating pain transmission.^[47]^ IL-6 exacerbates trigeminal neuropathic pain by upregulating the mechanosensitive channel Piezo2 in trigeminal nociceptive neurons – heightening mechanical allodynia and pinprick hyperalgesia – whereas IL-6 neutralization mitigates these behaviors.^[48]^ Our findings now firmly suggest that IL-6 is not merely correlated with TN but causally contributes to its onset. This implies that a person with a genetic tendency toward higher IL-6 production may harbor a primed neuroinflammatory state that makes trigeminal nerve insults (such as vascular compression) more likely to produce neuralgia. Targeting IL-6 signaling, therefore, could be a rational approach to prevent or ameliorate TN flares – a concept supported by the success of IL-6R antagonists in other inflammatory conditions.^[49]^

Neurturin is a more unexpected risk factor for TN. It is a member of the (glial cell line–derived neurotrophic factor) family and primarily signals through the GFRα2/RET receptor complex to support the survival and function of certain populations of peripheral neurons.^[50]^ Paradoxically, while neurotrophic factors generally promote neuron growth and survival, excessive or misdirected neurotrophic signaling can lead to aberrant nerve sprouting or sensitization of pain fibers.^[51]^ Our MR results indicate that genetically higher neurturin levels increase TN risk, suggesting that neurturin-related pathways may facilitate pathological rewiring or hyperexcitability of trigeminal nerve circuits. Preclinical evidence from pain models substantiates this idea. In models of bone cancer pain and inflammatory bone pain, neurturin acting on GFRα2-expressing nociceptors was shown to induce hyperalgesia, and sequestration (neutralization) of neurturin or knockout of GFRα2 significantly reduced pain behaviors.^[52]^ Neurturin predominantly affects nonpeptidergic (IB4-positive) C-fiber neurons; in the trigeminal system, an overabundance of neurturin could thus enhance sprouting or neurotransmitter release from this subset of pain fibers, contributing to heightened pain sensitivity. Supporting a role in human pain, a clinical study in pancreatic cancer patients noted that upregulation of neurturin and its receptor (GFRα2) in intrapancreatic nerves was associated with more severe pain phenotypes.^[53]^ Taken together, these data suggest that an overactive neurturin/GFRα2 axis might render trigeminal sensory pathways hyper-innervated or hyper-responsive, thereby increasing the likelihood of TN. Our finding is the first to implicate neurturin in TN, broadening the scope of TN’s etiology to include dysregulated neurotrophic support.

In practical terms, IL-6 and neurturin represent upstream “triggers” in TN pathogenesis. IL-6 exemplifies how systemic or CNS inflammation can lower the threshold for central sensitization and neuropathic pain, while neurturin points to developmental or regenerative signaling processes that, when overactive, become maladaptive. These insights encourage a broader view of TN as not solely a focal compression neuropathy but as a condition influenced by an individual’s proinflammatory status and neurotrophic milieu. Therapies that dampen IL-6 (for instance, IL-6R blockers like tocilizumab) or modulate GDNF-family signaling (e.g., GFRα2 antagonists or ligand traps) might thus reduce the risk or severity of TN in genetically susceptible patients – an avenue that warrants further investigation in future studies.

### 4.3. TN’s downstream immune effects: IL-15RA and TGF-α

Finally, our results shed light on immune changes that appear to be consequences of TN rather than causes. Specifically, we found that TN had a unidirectional causal effect in raising the levels of IL-15RA and TGF-α, whereas genetically determined variation in these factors did not influence TN risk. This pattern suggests that TN’s pathology and pain trigger secondary immune responses marked by these molecules.

IL-15RA is the high-affinity receptor subunit that binds IL-15, a cytokine important for activating natural killer cells, T cells, and microglia.^[54,55]^ While IL-15 itself did not emerge as a primary causal trigger for TN in our analysis, the reverse causal link implies that TN episodes drive an upregulation of IL-15/IL-15RA signaling.^[35]^ This could reflect a reactive immune activation in the trigeminal system: severe axonal injury and pain may stimulate resident glial cells and infiltrating macrophages to produce IL-15, which in turn promotes further immune cell recruitment or survival in the affected area.^[56]^ Notably, IL-15 has been identified as one of the key early inflammatory mediators released after peripheral nerve injury.^[57]^ It can contribute to pain hypersensitivity by enhancing nociceptor excitability and sustaining microglial activation. For example, IL-15 can promote immune system activation and nociceptor sensitization in headache disorders such as migraine,^[58]^ suggesting it may sensitize trigeminal pain pathways. Moreover, prior studies have shown that IL-15 (and IL-15RA) levels are elevated in certain inflammatory joint diseases and correlate with pain severity; in osteoarthritis and rheumatoid arthritis, IL-15 signaling has been linked to symptomatic pain, and blocking IL-15 can alleviate pain in clinical trials.^[59]^ In the context of TN, the observed elevation of IL-15RA likely indicates that chronic trigeminal nerve distress engages an IL-15–mediated loop aimed at immune defense or tissue repair – potentially by mobilizing T cells or sustaining microglial reactions in the trigeminal nucleus. This response might be a double-edged sword: on one hand, IL-15 could help clear debris or promote remyelination, but on the other hand, excessive IL-15 signaling may also perpetuate neuroinflammation and pain.

TGF-α is a growth factor and a ligand of the epidermal growth factor receptor (EGFR).^[60]^ Although the role of TGF-α in pain mechanisms has been less extensively studied, existing evidence links EGFR signaling to neuropathic pain.^[61]^ Following nerve injury, reactive glial cells – particularly astrocytes – upregulate EGFR ligands such as amphiregulin and TGF-α, as part of gliosis and wound-healing responses.^[62]^ Recent studies indicate that EGFR activation in astrocytes contributes to the maintenance of neuropathic pain, and pharmacological blockade of EGFR reduces both pain behavior and astroglial reactivity.^[63]^ Mechanistically, TGF-α released from neurons or glia can activate EGFR on satellite glia or macrophages,^[64]^ inducing CCL2 production and recruiting CCR2⁺ monocytes that amplify nociceptive signaling.^[65]^ Consistent with this model, our MR analysis indicates that genetic liability to TN is positively associated with higher circulating TGF-α levels. Thus, elevated TGF-α in TN likely reflects ongoing glial and immune activation in response to trigeminal nerve injury – initially reparative but capable of sustaining pain via EGFR-dependent signaling. Importantly, TGF-α may not initiate TN; once TN is established (with demyelination and axonal stress), TGF-α becomes one of several upregulated mediators that reinforce central sensitization. This interpretation aligns with clinical findings in other painful conditions. For example, in oral and bone cancers, increased expression of EGFR ligands (including TGF-α) has been reported in affected tissues, and EGFR inhibitors are currently being investigated as non-opioid analgesics for cancer-related pain.^[65]^ Our results therefore suggest a parallel mechanism in TN: disease-driven upregulation of EGFR ligands such as TGF-α may sustain pain by reinforcing astrocyte–EGFR signaling.

We employed a bidirectional 2-sample MR framework to evaluate the causal relationships between circulating inflammatory proteins and TN, and further stratified the results into “bidirectional–forward–reverse” analyses to facilitate the identification of potentially translatable pathways and biomarkers. Nonetheless, several limitations merit consideration: MR estimates capture lifelong, genetically proxied exposure and may miss short-term dynamics or non-linear/threshold effects; despite using multiple complementary estimators, residual horizontal pleiotropy, incomplete LD colocalization, and sample overlap/winner’s curse may still bias effect sizes; variability in TN case definitions and subtyping (e.g., classical vs secondary forms, neurovascular compression status) and in ancestry structure introduces heterogeneity that may constrain generalizability across populations; and MR findings do not readily translate into actionable clinical thresholds or pharmacologic dosing. Future work should replicate and colocalize key loci in multi-ancestry prospective cohorts, integrate paired plasma–CSF multi-omics with functional experiments to validate pathways, assess temporal causality and dose–response relationships, and pursue mechanism-driven interventional studies to establish clinical utility.

## 5. Conclusions

In conclusion, our comprehensive bidirectional 2-sample MR framework demonstrated that circulating immune factors are causally linked to TN. We identified bidirectional associations for eotaxin (CCL11), CXCL5, and IL-20RA, supporting a neuro-immune feedback loop, while forward MR indicated that higher IL-6 and Neurturin increase TN risk, and reverse MR showed that TN lowers circulating IL-15RA and TGF-α. These findings, robust across complementary estimators and sensitivity analyses, highlight translatable pathways (IL-20/IL-20RA, CXCL5, IL-6, Neurturin) and candidate peripheral indicators (IL-15RA, TGF-α) for risk stratification and therapeutic targeting in TN. Our genetic evidence demonstrates a close coupling between the circulating inflammatory network and TN, providing testable leads for biomarker development and immunomodulatory strategies in TN.

## Acknowledgments

Thanks to all authors for their contributions.

## Author contributions

**Conceptualization:** Lingsen Hou, Jinzhou Guo, Shanshan Cui.

**Data curation:** Lingsen Hou, Lingbo Hou, Jinzhou Guo.

**Formal analysis:** Lingsen Hou, Jinzhou Guo, Shanshan Cui.

**Funding acquisition:** Shanshan Cui.

**Investigation:** Lingsen Hou, Jianwen Guo.

**Methodology:** Lingbo Hou.

**Project administration:** Lingbo Hou, Jianwen Guo, Shanshan Cui.

**Resources:** Jianwen Guo.

**Software:** Lingsen Hou, Lingbo Hou, Jinzhou Guo.

**Supervision:** Jianwen Guo, Jinzhou Guo, Shanshan Cui.

**Validation:** Lingsen Hou, Lingbo Hou.

**Visualization:** Lingsen Hou, Lingbo Hou, Jinzhou Guo.

**Writing – original draft:** Lingsen Hou.

**Writing – review & editing:** Lingbo Hou, Jianwen Guo, Shanshan Cui.

## Supplementary Material




