# Supplementary material for: Causal interplay between trigeminal neuralgia and systemic inflammatory markers: A bidirectional Mendelian randomization study
Source: Medicine (Baltimore). 2026 Jan 23;105(4):e47243. doi: 10.1097/MD.0000000000047243 (PMC12851747; doi:10.1097/MD.0000000000047243)

**Supplementary figure 1**: MR leave-one-out sensitivity analysis of causal relationships between 5 circulating inflammatory proteins and TN.


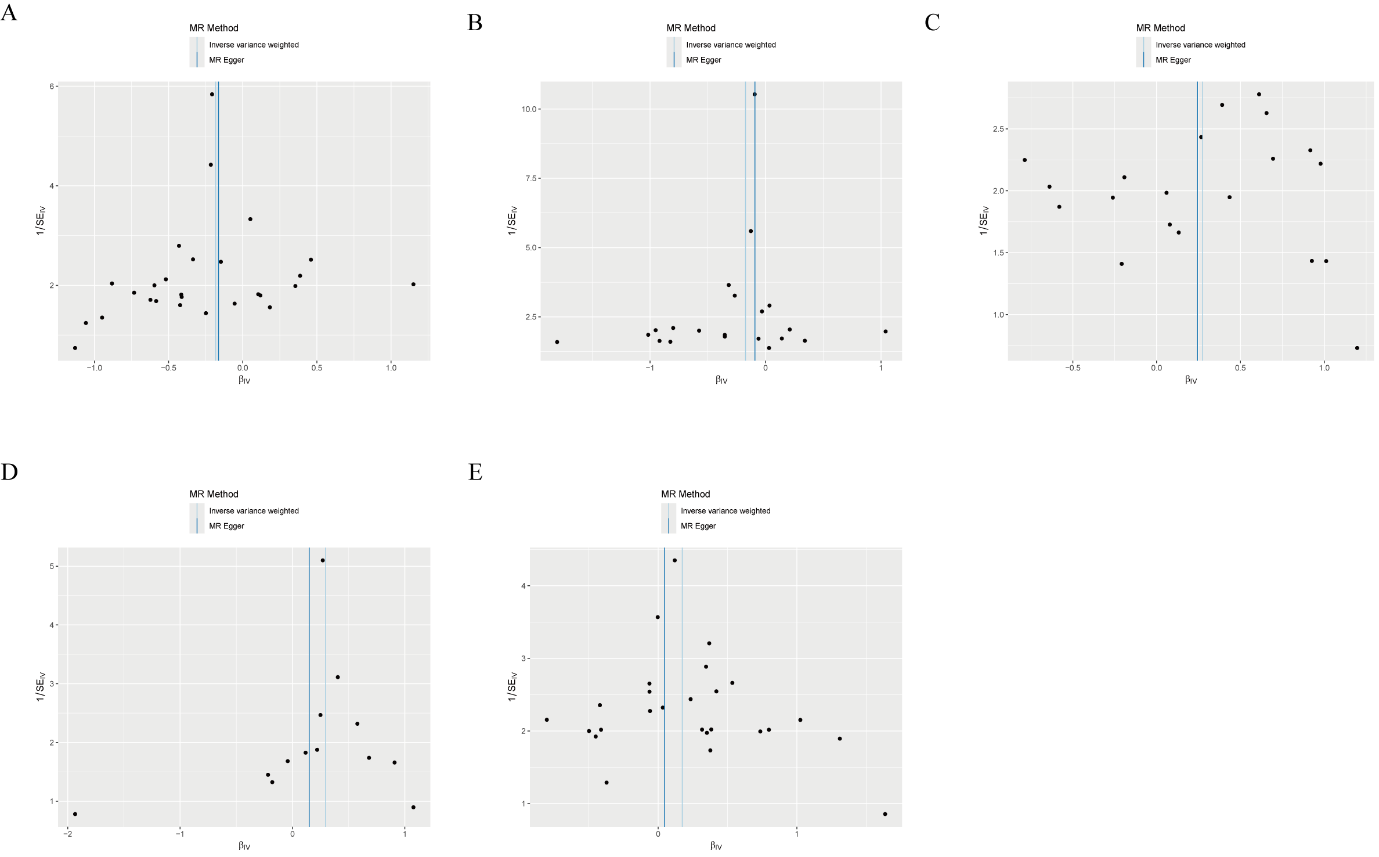


**Supplementary figure 2**: Funnel plot to assess heterogeneity of causal relationships between 5 circulating inflammatory proteins and TN.


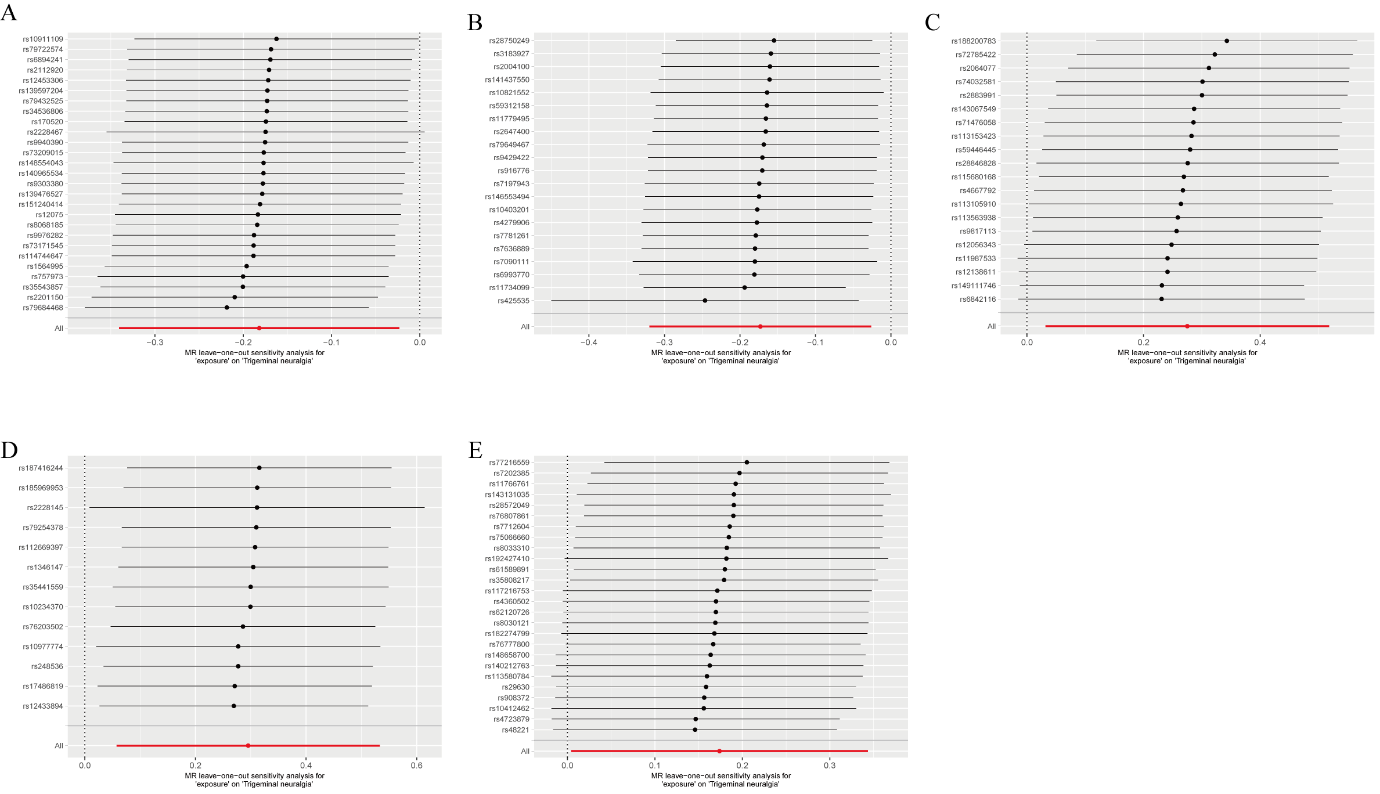


**Supplementary figure 3**: MR leave-one-out analysis of causal relationships between 5 circulating inflammatory proteins and TN.


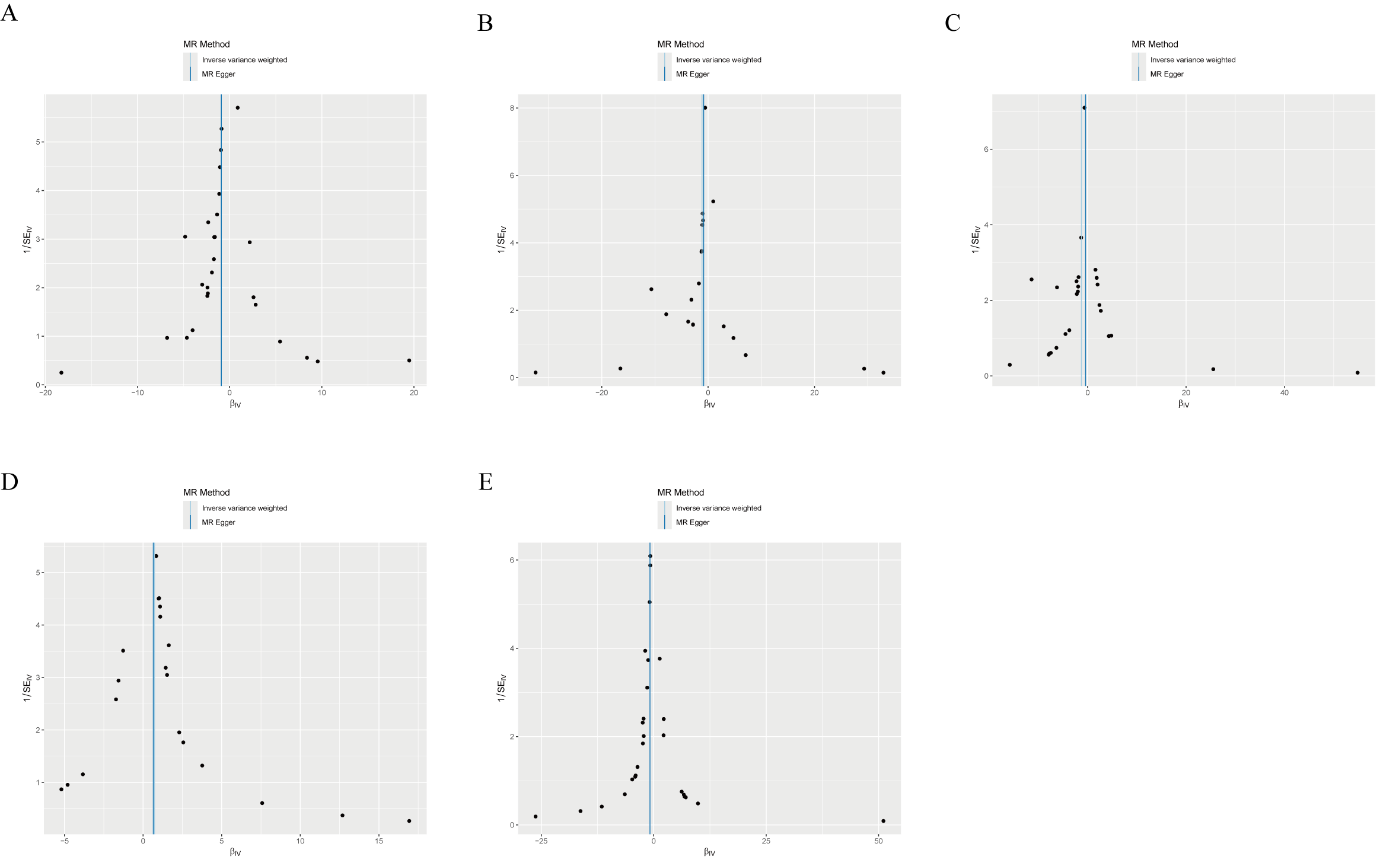


**Supplementary figure 4**: Funnel plot to assess heterogeneity of causal relationships between 5 circulating inflammatory proteins and TN.


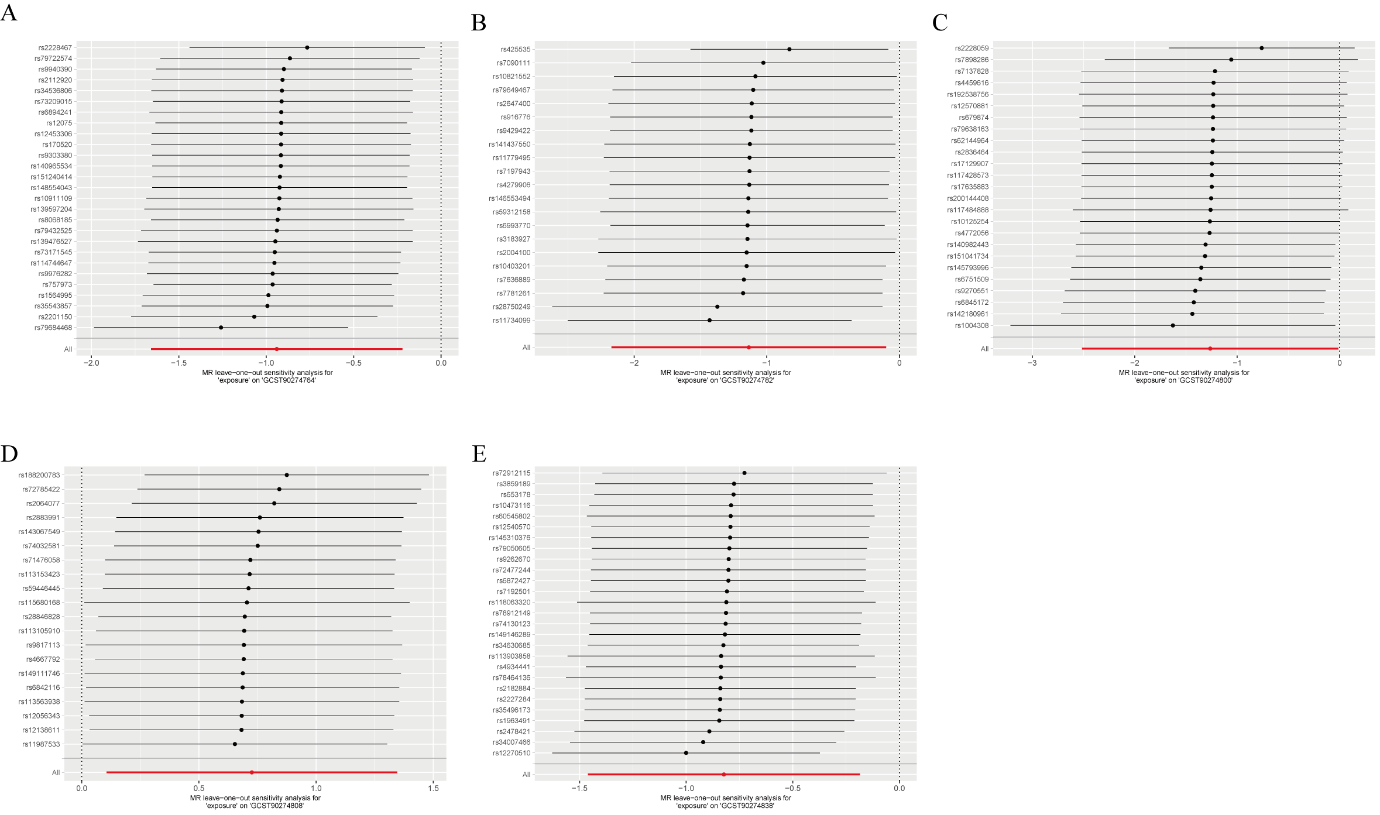

Supplement: Supplementary file 2 [file medi-105-e47243-s002.docx]
